# Cross talk of signals between EGFR and IL-6R through JAK2/STAT3 mediate epithelial–mesenchymal transition in ovarian carcinomas

**DOI:** 10.1038/sj.bjc.6604794

**Published:** 2008-12-16

**Authors:** M Colomiere, A C Ward, C Riley, M K Trenerry, D Cameron-Smith, J Findlay, L Ackland, N Ahmed

**Affiliations:** 1 Women's Cancer Research Centre, Royal Women's Hospital, Melbourne, Australia; 2 Centre for Cellular and Molecular Biology, Deakin University, Melbourne, Australia; 3 School of Medicine, Deakin University, Melbourne, Australia; 4 School of Exercise and Nutrition Sciences, Deakin University, Melbourne, Australia; 5 Prince Henry's Institute of Medical Research, Melbourne, Australia; 6 Department of Obstetrics & Gynaecology, University of Melbourne, Melbourne, Australia; 7 Department of Surgery, University of Melbourne, Melbourne, Australia

**Keywords:** ovarian carcinoma, epithelial–mesenchymal transition, migration, Janus kinase 2, signal transducer and activator of transcription 3

## Abstract

Epidermal growth factor receptor (EGFR) is overexpressed in ovarian carcinomas, with direct or indirect activation of EGFR able to trigger tumour growth. We demonstrate significant activation of both signal transducer and activator of transcription (STAT)3 and its upstream activator Janus kinase (JAK)2, in high-grade ovarian carcinomas compared with normal ovaries and benign tumours. The association between STAT3 activation and migratory phenotype of ovarian cancer cells was investigated by EGF-induced epithelial–mesenchymal transition (EMT) in OVCA 433 and SKOV3 ovarian cancer cell lines. Ligand activation of EGFR induced a fibroblast-like morphology and migratory phenotype, consistent with the upregulation of mesenchyme-associated N-cadherin, vimentin and nuclear translocation of *β*-catenin. This occurred concomitantly with activation of the downstream JAK2/STAT3 pathway. Both cell lines expressed interleukin-6 receptor (IL-6R), and treatment with EGF within 1 h resulted in a several-fold enhancement of mRNA expression of IL-6. Consistent with that, EGF treatment of both OVCA 433 and SKOV3 cell lines resulted in enhanced IL-6 production in the serum-free medium. Exogenous addition of IL-6 to OVCA 433 cells stimulated STAT3 activation and enhanced migration. Blocking antibodies against IL-6R inhibited IL-6 production and EGF- and IL-6-induced migration. Specific inhibition of STAT3 activation by JAK2-specific inhibitor AG490 blocked STAT3 phosphorylation, cell motility, induction of N-cadherin and vimentin expression and IL6 production. These data suggest that the activated status of STAT3 in high-grade ovarian carcinomas may occur directly through activation of EGFR or IL-6R or indirectly through induction of IL-6R signalling. Such activation of STAT3 suggests a rationale for a combination of anti-STAT3 and EGFR/IL-6R therapy to suppress the peritoneal spread of ovarian cancer.

Despite considerable progress achieved in the management of gynaecological cancers, cure of epithelial ovarian cancer still remains evasive, with an overall 5-year mortality of 70%. The peritoneal spread of ovarian cancer to surrounding organs within the abdomen relies on the movement of cancer cells, which are regulated in an autocrine or a paracrine manner by growth factors and cytokines present in the peritoneal tumour fluid (Puiffe *et al*, 2007). In this context, the contribution of epithelial–mesenchymal transition (EMT) by endogenous or exogenous growth factors may be vital to initiate and sustain abdominal cell motility of tumour cells. Recently, some studies have reported EMT-like processes in ovarian cancer cells in response to stimuli present in the ascites (Theriault *et al*, 2007), but the exact role of the process in the context of cancer progression still remains elusive.

We have previously shown that ovarian surface epithelial cells (Ahmed *et al*, 2006) and ovarian cancer cells undergo EMT in response to EGF (Ahmed *et al*, 2007; Lim *et al*, 2007). Elevated expression of epidermal growth factor receptor (EGFR) and its ligand are common in many epithelial cancers including ovarian cancer (Psyrri *et al*, 2005). Epidermal growth factor/EGFR autocrine and paracrine processes driving tumour cell proliferation has formed the basis for chemotherapeutic drug targeting of EGFR in some cancers (Blank *et al*, 2005). Activated EGFR triggers diverse signalling pathways in tumour cells (Ahmed *et al*, 2006; Yarden and Shilo, 2007). We have recently shown that EGF-induced EMT in ovarian surface epithelial cells is associated with matrix remodelling through matrix metalloproteases and is mediated by the classical extracellular signal-regulated kinase (ERK) and integrin-linked kinase (ILK)/glycogen synthase kinase 3*β* pathways (Ahmed *et al*, 2006). Integrin-linked kinase pathway is also responsible for EMT in ovarian cancer induced by endothelin, and combined targeting of endothelin receptor and EGF receptors has shown enhanced antitumour activity in ovarian cancers (Bagnato and Rosanò, 2007; Rosanò *et al*, 2007). Beside endothelin, bone morphogenetic proteins induce EMT-like changes in ovarian cancer cells (Theriault *et al*, 2007). In addition, VEGF and lysophosphatidic acid, which are present in high abundance in the ascites and serum of ovarian cancer patients, induces invasiveness in cultured ovarian cancer cell lines (Ahmed *et al*, 2006; Wang *et al*, 2006). In a recent study, we have shown that ascites from ovarian cancer patients induce distinct changes associated with invasiveness in ovarian cancer cells (Ahmed *et al*, 2005). However, whether this facilitation of invasiveness is EMT-dependent still remains elusive.

Janus kinase (JAK)/signal transducer and activator of transcription (STAT) signalling is required for diverse processes during embryogenesis (Teng *et al*, 2004), and activation of this pathway has been associated with cancers (Chen *et al*, 2007). This pathway is activated when an extracellular ligand binds to its receptor resulting in the activation of JAK(s) and phosphorylation of specific tyrosine residues on the receptor. Signal transducer and activator of transcription proteins are recruited to the receptor by binding to the phosphorylated tyrosine residues through the SRC homology domain 2 in the STAT proteins. Signal transducer and activator of transcriptions are then phosphorylated by JAK, after which they dimerise and shuttle to the nucleus where they function as a transcription factor. Notably, interleukin (IL)-6, oncostatin M and leukaemia inhibitory factor (LIF) signal through receptors that share a common GP130 receptor subunit, which signals through JAK2 and STAT3 (Carbia-Nagashima and Arzt, 2004). Signal transducer and activator of transcription3 activation can also occur through EGFR either by direct ligand activation or by indirect ligand-independent transactivation, and such activation have been shown to mediate cancer cell proliferation and carcinogenesis (Sriuranpong *et al*, 2003; Chan *et al*, 2004).

In humans, STATs have a unique expression pattern and distinct physiological functions (Levy and Darnell, 2002). Signal transducer and activator of transcription3 has been shown to maintain cancer cell proliferation, as inhibition of STAT3 led to inhibition of growth in several cancer cell lines (Chen *et al*, 2007), including ovarian cancer (Silver *et al*, 2004). However, naturally occurring mutations in STAT3 protein resulting in its constitutive activation have not been identified, suggesting that aberrant growth factor signalling, frequent in cancer, may regulate the constitutive activation of STAT3.

Recent evidence indicates a role for STAT3 in cell motility. In zebrafish embryos undergoing gastrulation, STAT3 is essential for the migration of sheets of cells (Yamashita *et al*, 2002). STAT3 is also required for mouse gastrulation, which represents a classical EMT model in embryonic development (Takeda *et al*, 1997). Mouse keratinocytes conditionally depleted of STAT3 show defects in wound healing (Sano *et al*, 1999). Activation of JAK/STAT pathway in the *Drosophila* ovary causes the ‘extra cells’ to become invasive, while the migration of a subset of epithelial follicular cells called the ‘border cells’ is dependent on STAT expression (Silver and Montell, 2001). As initiation of the human ovarian cancer postulated to originate from the ovarian surface epithelium with subsequent exfoliation of tumour cells in the peritoneum somewhat resembles the situation in the *Drosophila* ovary, it is not surprising that several of the genes that control *Drosophila* epithelial follicle cell migration are homologous to genes implicated in the progression of ovarian cancer (Montell, 2003).

In addition to the migratory role of STAT3 described above, recent studies have reported an association between STAT3 and EMT (Lo *et al*, 2007). This is mediated through E-cadherin repressors, the zinc-finger transcription factor Snail and estrogen-regulated zinc transporters LIV-1 in breast tumours (Taylor *et al*, 2004). Recently, gastrin has been reported to induce EMT in colon cancer through the JAK2/STAT3 pathway (Ferrand *et al*, 2004), while gastrin-releasing peptides have been shown to transactivate EGFR in pancreatic cancer (Thomas *et al*, 2005). In addition, oncostatin M has been shown to induce EMT in tubular epithelial cells through GP130-mediated activation of JAK2/STAT3 pathway (Nightingale *et al*, 2004), indicating that this pathway may be critical for cytokine and growth factor-mediated responses regulating EMT biology in fibrogenesis and cancer.

In the light of the evidence supporting the activated status of STAT3 in ovarian carcinomas and the role of STAT3 in cell motility and EMT, this study sought to directly examine the role of this pathway in EGF-induced EMT in ovarian cancer cells. Although tyrosine kinase receptors, such as EGFR, are capable of activating STAT3, the incidence of STAT3 activation by inflammatory cytokines (such IL-6) in cooperation with EGFR is common among some tumours (Badache and Hynes, 2001). Thus, it is possible that additional pathways other than EGFR may participate in the activation of STAT3 to induce EMT. To investigate that, we analysed the activation status of JAK2/STAT3 in ovarian carcinoma specimens and correlated that to STAT3 activation in two ovarian cancer cell lines directly by EGFR or indirectly through IL-6R activation. We studied the association between the increased levels of STAT3 activation and high-grade ovarian carcinomas, consistent with a role for STAT3 in the motile phenotype of ovarian cancer cells in response to direct activation by EGFR or indirectly through IL-6R. In addition, we report that the JAK2/STAT3 pathway is required to sustain EGF-induced EMT-associated phenotypes in ovarian cancer cells, indicating that in EGF-driven ovarian tumours JAK2/STAT3 signalling may be critical in regulating metastasis.

## Materials and methods

### Cell lines

The human epithelial ovarian cancer lines OVCA 433 and SKOV3 were obtained from Dr Robert Bast, MD Anderson Centre, Houston, USA and have been described previously (Ahmed *et al*, 2003). Cell lines were grown as monolayers in 25 or 75 cm^2^ flasks (Nunclon, Roskilde, Denmark) in complete growth medium consisting of 50% medium 199 (Sigma-Aldrich, Sydney, Australia) and 50% MCDB105 (Sigma-Aldrich) supplemented with 10% (v/v) heat inactivated fetal bovine serum and 2 mM glutamine (Invitrogen Corporation, Melbourne, Australia) in the presence of 37°C with 5% CO_2_.

### Antibodies and reagents

Monoclonal and polyclonal antibodies against phosphorylated (P)-ERK1/2, total (T)-ERK1/2, (P)-JAK2, T-JAK2, P-STAT3 (Tyr 705) and T-STAT3 were obtained from Cell Signalling Technology (Beverly, MA, USA). Monoclonal antibodies against vimentin and N-cadherin were obtained from Zymed Laboratories (San Francisco, CA, USA). Polyclonal antibody against *β*-catenin was obtained from Sigma-Aldrich and anti-human IL-6R neutralising antibody was from R&D Systems (Minneapolis, MN, USA). The secondary antibodies were purchased from Chemicon International (Temecula, CA, USA) and Molecular Probes (Eugene, OR, USA). Epidermal growth factor and AG490 were obtained from Chemicon International and Calbiochem (San Diego, CA, USA), respectively.

### Tissues

The study was approved by the Research and Human Ethics Committee (HEC no. 02/30) of The Royal Women's Hospital, Melbourne, Australia. Ovarian cancer patients with serous, mucinous, endometrioid, clear cell carcinoma and mixed subtypes were included in the study. The histopathological diagnosis and tumour grades were determined by staff pathologists as part of the clinical diagnosis. Histological grading of ovarian carcinoma was determined by the method described by Silverberg (2000). Normal ovaries were removed from patients undergoing surgery as a result of suspicious ultrasound images, palpable abdominal masses and/or family history. Archival tissues were obtained from the Department of Pathology, Royal Women's Hospital, from women who presented for surgery after the provision of a participant information statement and with informed consent. The mean age of healthy volunteers and women presenting with benign and borderline tumours was 57 years. The mean age of women with grades 1, 2 and 3 cancer was 58 years.

### Description of women included in the JAK2/STAT3 study

With few exceptions common tissues were used for JAK2, STAT3 and P-STAT3 expression. A total of 58 women were included in the study. Among these tissues nine were from normal women, ten in each group were from women with benign, borderline, grades 1 and 2 tumours, respectively and nine were from grade 3/4 patients. Among the grade 1 patients, 80% had stage 1 disease, while the rest 20% had stages 2 and 3. On the other hand, 20–30% of grade 2 patients had stage 1 cancer, while 70–80% had stage 3/4. Similarly, 80–90% of grade 3 patients were diagnosed at stage 3/4, while 10–20% had stages 1 or 2 diseases.

A limited number of normal, benign, borderline, grades 1, 2 and 3/4 tissues (*n*=14) of serous, mucinous and clear cell subtype were evaluated for P-JAK2 status.

Immunohistochemical analysis on ovarian tissues was performed as described previously (Ahmed *et al*, 2002a, 2002b, 2003). Briefly, paraffin sections were cut at 4 *μ*m thickness, mounted on silane-coated slides and incubated overnight at 37°C. Sections were washed with water after two changes of xylene and three changes of ethanol. Antigen retrieval was performed using citrate buffer (pH 6.0), and sections were held in Tris-buffered saline. Endogenous peroxidase activity was removed using 3% hydrogen peroxide in methanol. The sections were incubated for 1 h in primary antibody diluted 1/200 in 1% BSA in Tris buffer (100 mM, pH 7.6). Antibody binding was amplified using biotin and streptavidin HRP (Chemicon International) for 15 min each, and the complex was visualised using diaminobenzidine (DAB). Nuclei were lightly stained with Mayer's haematoxylin. Non-immune rabbit serum was used as a control. The specificity of the antibodies was also evaluated by western blot in ovarian cancer cell lysates. In all cases right molecular weight bands were observed.

Sections were assessed microscopically for positive DAB staining. Two observers (NA and CR) independently evaluated the immunostaining results and the degree of staining was scored in a blind manner. The concordance ratio was >95% in each case. Differences in opinion were resolved by re-evaluating the sections and in some cases by reaching a consensus with the assistance of a third evaluator. Four sections were assessed per tissue and tissue and cellular distribution of staining was determined. For each specimen, the positive staining extent was scored in five grades, namely: 0 (⩽10%), 1 (⩾11–25%), 2 (⩾26–50%), 3 (⩾51–75%), 4 (⩾76–90%) and 5 (⩾90–100%).

### SDS–PAGE and western blot analyses

Western blot and SDS–PAGE were performed on cell lysates by the methods described previously (Ahmed *et al*, 2003). Protein loading was monitored by stripping the membrane with Re-Blot solution (Chemicon International) and re-probing the membrane with *β*-actin primary antibody (Sigma-Aldrich). Densitometry (OD mm^−2^) was performed using the Multi Gauge V2.3 computer program.

### Preparation of serum-free medium and IL-6 sandwich ELISA

Cells were grown in 25 cm^2^ flask in standard growth medium until 80–90% confluence was attained. Cells were washed in serum-free medium three times and incubated in the same medium for 24 h. The medium was collected by centrifugation, and protein content in the medium was estimated using a commercial protein assay kit with BSA standards according to the manufacturer's instructions (Pierce, Rockford, IL, USA). The IL-6 sandwich ELISA was performed using a sandwich ELISA kit (Biosource International, Camarillo, CA, USA) according to the manufacturer's instruction. All data were normalised for total protein and expressed as pg mg^−1^ of protein.

### Indirect immunofluorescence

Immunofluorescence analysis of N-cadherin, *β*-catenin and vimentin was performed as described previously (Lebret *et al*, 2007). Images were captured by the photo multiplier tube using the Leica TCS SP2 laser, and were viewed on an HP workstation and the Leica microsystems TCS SP2 software.

### Cell migration assay

Cell migration assay was performed as described previously (Lebret *et al*, 2007). Photos were taken of the scratches at 0 and 14 h using a Sony Digital Camera. The width of the scratches was measured at each time point and quantified using Microsoft Excel computer program.

### RNA extraction and RT–PCR

Total cellular RNA was extracted and analysed by real-time RT–PCR as previously described (Trenerry *et al*, 2007). Primers (Supplementary Information) were designed using Primer Express software package version 3.0 (Applied Biosystems, Foster city, CA, USA) from gene sequences obtained from GenBank and checked for specificity using nucleotide–nucleotide Blast search. The efficacy of cyclophilin as endogenous controls was examined using the Equation 2^−ΔCt^.

### Statistical analysis

The significance of the extent of immunohistochemical staining between normal, benign, borderline, grades 1, 2 and 3 ovarian tumours was determined by the non-parametric *χ*^2^ contingency test (Ahmed *et al*, 2002a, 2002b). Student's *t*-test was also used for the statistical analyses of migration assay and western blot data. Data are presented as mean±standard error of the mean (s.e.m.). Each experiment was repeated at least three times in triplicate. Statistical analysis for RT–PCR was performed using GraphPad Prism 4.1 (GraphPad Software, San Diego, CA, USA) (Trenerry *et al*, 2007). Means were compared using two-way ANOVA and any significant differences were analysed using a Bonferroni *post hoc* test. Data are presented as mean±s.e.m. A probability level of *P*<0.05 was adopted throughout to determine statistical significance unless otherwise stated.

## Results

### JAK2/STAT3 status in ovarian carcinomas

**Figure 1:**
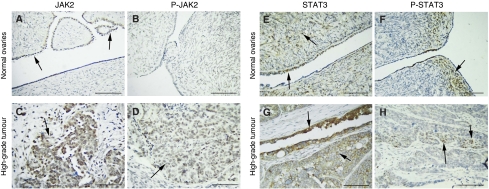
Activation of JAK2 and STAT3 in ovarian carcinomas. Expression of JAK2, P-JAK2, STAT3 and P-STAT3 in archival normal ovaries and high-grade ovarian tumours using the immunoperoxidase method, as discussed in the Materials and Methods. (**A**) Normal ovary, arrows indicating continuous expression of JAK2 in the epithelium. (**B**) No expression of P-JAK2 was evident in the stroma or epithelium of the normal ovary. (**C** and **D**) Scattered cytoplasmic and membrane staining of JAK2 and P-JAK2 (indicated by arrows) in a serous high-grade ovarian tumour. Same tissues were used to evaluate the expression of JAK2 and P-JAK2. (**E** and **F**) Nuclear and cytoplasmic expression of STAT3 and P-STAT3 (indicated by arrows) in the epithelium and stroma of normal ovaries. (**G** and **H**) Epithelial staining of P-STAT3 (nuclear and cytoplasmic) in an endometrioid high-grade ovarian carcinoma. Magnification × 40, scale=50 *μ*m.

We compared the expression and activation status of JAK2 and STAT3 in a range of benign, borderline, and malignant ovarian tumours and normal ovaries, using immunohistochemistry (Figure 1). Expression of JAK2 was observed in the epithelial cells and the stroma of all samples. Both cytoplasmic and membrane-associated JAK2 expression was significantly different between the normal ovaries and the tumour groups (Figure 1A and C) (Table 1). In contrast, activated JAK2, as detected using P-JAK2-specific antibodies, was observed only in the epithelium of grades 1 and 3 tumours and was not present in the normal, benign and borderline tumours (Figure 1B and D). Signal transducer and activator of transcription3 was expressed in both epithelial and stromal cells, with no significant difference observed between normal ovaries, benign, borderline and malignant tumours (Figure 1E and G). However, there was a significant difference in the levels of activated STAT3 between the normal, benign, borderline and histological grades of tumours (Table 1), with latter showing the highest levels of expression (Figure 1F and H). P-STAT3 was observed in both the nucleus and cytoplasm. None of the tissues showed any positive staining with the control IgG antibodies.

### EGF-induced changes in the expression and localisation of N-cadherin, vimentin and *β*-catenin and also increased motility of ovarian cancer cells

**Figure 2:**
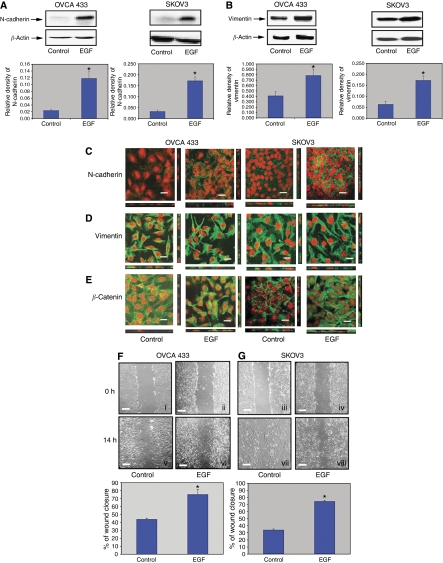
(**A** and **B**) Enhanced expression of N-cadherin and vimentin by EGF. Cell lysates were prepared and western blot analysis was performed as described in the Materials and Methods. Blots were stripped and re-probed with *β*-actin to indicate relative amounts of protein loaded. Three independent experiments were performed and each blot is a representative of one individual experiment. Graphs are represented as the mean density of N-cadherin and vimentin bands normalised against the mean density of *β*-actin band from three independent experiments (presented as relative density of individual protein). ^*^P<0.05 compared to control untreated cells. (**C**–**E**) Expression and localisation of N-cadherin, vimentin and *β*-catenin in OVCA 433 and SKOV3 cell lines in the presence and absence of EGF. Confocal microscopy was performed as described in the Materials and Methods. Proteins were visualised by Alexa 488 fluorescent label (green) and nuclei were visualised by ethidium bromide staining (red). The image is a representative of four independent experiments. Scale=30 *μ*m. (**F** and **G**) Effect of EGF on the migration of OVCA 433 and SKOV3 cells. Cells were cultured on glass coverslips in either normal growth medium (control) or EGF-treated medium (10 ng ml^−1^). Using scratch tests over 14 h, SKOV3 and OVCA 433 cells were analysed for the extent of wound closure as described in the Materials and Methods. For each coverslip four scratches were made and the width of each scratch was measured at four different locations at 0 and 14 h time points to determine the extent of wound. Phase contrast images are representative of one independent experiment repeated three times. The graphs represent mean % of wound closure from three independent experiments. ^*^P<0.05 compared to control untreated cells. Scale=300 *μ*m.

The relationships between the observed activation of the JAK2/STAT3 pathway in ovarian specimens were correlated with EGF-induced phenotypic changes in OVCA 433 and SKOV3 ovarian carcinoma cell lines. These cells typically grow as epithelial cobblestone-like monolayers but show a fibroblast-like morphology typical of mesenchymal cells in the presence of EGF (Lim *et al*, 2007). Epidermal growth factor-treatment of OVCA 433 and SKOV3 cells lead to a significant increase in both N-cadherin and vimentin expression within 24 h (Figure 2A and B) (Colomiere *et al*, 2008). However, EGF-induced EMT had no significant effect on *β*-catenin expression in either cancer cell line (data not shown).

Immunofluorescence studies revealed cell–cell junction localisation of N-cadherin in control untreated cells. Within 24 h of EGF treatment, concurrent with the enhanced expression of N-cadherin, some granular cytoplasmic localisation of the protein was observed in addition to the cell–cell junction localisation (Figure 2C). In contrast, the localisation of vimentin in both control and EGF-treated cells remained filamentous (Figure 2D). However, EGF treatment resulted in the translocation of *β*-catenin from the cytoplasm and cell junctions to discrete regions of the nucleus in certain population of cells (Figure 2E), consistent with the activation of the *β*-catenin-mediated pathways in response to EGF-associated EMT events (Polette *et al*, 2007).

Consistent with the changes in the expression and localisation of N-cadherin, vimentin and *β*-catenin, EGF also induced a motile phenotype in ovarian cancer cells as evaluated by wound-healing assay in serum-free medium (Ahmed *et al*, 2006; Lim *et al*, 2007). In the absence of EGF, 43% wound closure was observed in OVCA 433 cells after 14 h compared to 75% wound closure after EGF treatment under similar conditions, with essentially the same results obtained with SKOV3 cells (Figure 2F and G) (Colomiere *et al*, 2008). These results suggest that EGF enhances the EMT-associated motile phenotype of ovarian cancer cells.

### Activation of JAK2/STAT3 pathways induced by EGF correlates with EMT

**Figure 3:**
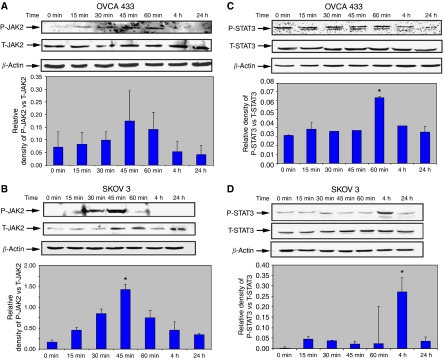
Activation of JAK2 and STAT3 in response to EGF. (**A**–**D**) Expression of activated and total JAK2 and STAT3 pre- and post-EGF treatment in OVCA 433 and SKOV3 cell lines. Cell lysates were prepared and western blot analysis was performed as described above. Experiments were performed three times and blots are representative of one independent experiment. Graphs are represented as the sum of the mean density of P-STAT3*α* and P-STAT*β* normalised against the mean density of T-JAK2 or T-STAT3 from three independent experiments (presented as relative density of phosphoprotein *vs* total protein). ^*^P<0.05 compared to control untreated cells at 0 min.

Epidermal growth factor is known to activate several signalling pathways (Yip *et al*, 2007), but direct activation of JAK2/STAT3 is rarely observed (Badache and Hynes, 2001). We investigate the potential involvement of this pathway in EGF-induced EMT. Epidermal growth factor stimulation lead to a significant induction of P-JAK2, which levels at 45 min (Figure 3A and B), and P-STAT3 peaking at 1 h in OVCA 433 and 4 h in SKOV3 cells (Figure 3C and D) relative to the expression of T-JAK2 and T-STAT3 (Colomiere *et al*, 2008). In OVCA 433 cells, active STAT3 showed double bands representative of *α-* and *β*-isoforms of the enzyme. The threshold activation of STAT3 in some ovarian cancer cell lines depended on the endogenous level of phosphorylated STAT3. OVCA 433 cells showed relatively higher level of endogenous active STAT3; hence, increment of active STAT3 in the presence of EGF was much less than SKOV3 cells. Moreover, in many cases the expression of phosphorylated proteins resulted in background noise, which in many experiments was difficult to avoid. This may be because of the low amount of phosphorylated proteins present in the cell lysates. No immunostaining was observed with control IgG antibodies.

### EGF induces the expression of IL-6 and LIF in ovarian cancer cell lines

**Figure 4:**
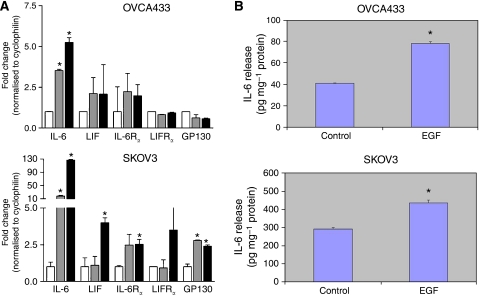
(**A**) Expression of ligands and receptor chains for the IL-6R and LIFR complexes following EGF stimulation. Gene expression was quantified using real-time PCR for each gene indicated before stimulation (open boxes) and then at 30 min (grey boxes) or 60 min (black boxes) following EGF stimulation of OVCA 433 (upper panel) or SKOV3 (lower panel). Values were normalised to the expression levels of the housekeeping gene cyclophilin, and expressed as a mean fold change from basal±s.e.m. Genes that showed a statistically significant increase in expression (*P*<0.05) are indicated by ^*^. (**B**) Quantification of IL-6 production by OVCA 433 and SKOV3 cells in the presence and absence of EGF. IL-6 secreted in the serum-free medium of the cell lines was measured by using the IL-6 sandwich ELISA kit. ^*^P<0.05 compared to control untreated cells.

To evaluate whether EGF would induce a secondary stimulus that may act in concert to stimulate the JAK2/STAT3 pathway, we used real-time RT–PCR to quantify the expression of IL-6 and LIF as well as their respective receptors. The levels of IL-6 mRNA were significantly increased following EGF stimulation of both OVCA 433 (5.2-fold, *P*<0.05) and SKOV3 (126-fold increase, *P*<0.05) cells (Figure 4). Leukaemia inhibitory factor mRNA was also increased, albeit to a lesser extent (OVCA 433, 2.1-fold, *P*<0.05; SKOV3, 4.0-fold). Importantly, the genes encoding the relevant receptor chains were expressed in each cell type: IL-6R*α* and GP130 constituting the IL-6 receptor complex and LIFR*α* and GP130, constituting the LIF receptor complex. Indeed, levels of the IL-6R*α* and GP130 mRNAs were modestly, but significantly increased following 1 h stimulation with EGF in SKOV3 cells (2.5- and 2.4-fold, respectively). These data support the idea that the observed EGF-induced activation of JAK2 and STAT3 may have resulted, at least in part, by the induction of IL-6 and LIF and subsequent autocrine activation by the common GP130 signal-transducing chains.

### EGF enhances the production of IL-6 in ovarian cancer cell lines

To determine if mRNA increase in IL-6R expression in response to EGF treatment has any affect on the production of IL-6 in ovarian cancer cell lines, we used IL-6 sandwich ELISA to quantify the amount of IL-6 secreted by OVCA 433 and SKOV3 cell lines in response to EGF. Both OVCA 433 and SKOV3 cell lines produced IL-6 endogenously but, in the presence of EGF, the production of IL-6 was increased (*P*<0.05) in the serum-free medium of both ovarian cancer cell lines (Figure 4B), consistent with the mRNA enhancement of IL-6 and IL-6R*α* on the cells (Figure 4A).

### Inhibition of EGF-induced IL-6 production and migration by neutralising IL-6R antibodies

**Figure 5:**
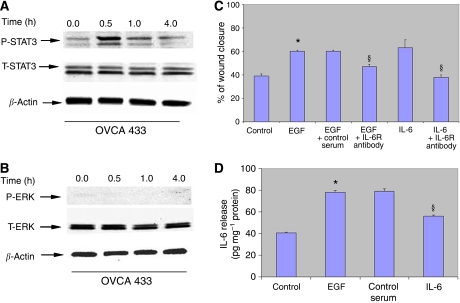
(**A** and **B**) Activation of STAT3 in response to IL-6 in OVCA 433 cells. Expression of P-STAT3 in response to IL-6 in OVCA 433 cell line. OVCA 433 cells were incubated in normal growth medium (control) or medium treated with IL-6 (20 ng ml^−1^) for 1 h. Western blot analysis was performed as described above for P-STAT3, P-ERK1/2 followed by re-probing first with T-STAT3, T-ERK1/2, respectively, followed by *β*-actin. (**C**) Effect of neutralising IL-6R antibody on the EGF or IL-6-induced migration in OVCA433 cells. Cells were cultured on glass coverslips in either normal growth medium (control) or EGF-treated medium (10 ng ml^−1^) or IL-6-treated medium (20 ng ml^−1^) in the presence and absence of IL-6R antibody (20 *μ*g ml^−1^). Using scratch tests over 14 h, cells were analysed for the extent of wound closure as described in the Materials and Methods. ^*^P<0.05, compared to control untreated cells, ^§^compared to cells treated with EGF or IL-6, respectively. (**D**) Effect of neutralising IL-6R antibody on IL-6 production in OVCA 433 cells. IL-6 production in the serum-free medium in the presence of +/−EGF and +/− anti-IL-6R or control serum was measured by sandwich ELISA assay as described in the Material and Methods. ^*^P<0.05, compared to control untreated cells, ^§^compared to cells treated with EGF.

We tested the functionality of the IL-6R complex by studying the response of OVCA 433 cells to IL-6. Strong activation of STAT3 was evident in OVCA 433 cells within 30 min, whereas no activation of classical ERK1/2 pathway was observed (Figure 5A and B). To evaluate the role of EGF-induced IL-6 synthesis on the migration in OVCA 433 cells, a wound-healing assay was performed in the presence of neutralising IL-6R antibody after treatment with IL-6 (20 ng ml^−1^) or EGF (10 ng ml^−1^). Both IL-6 and EGF induced migration in OVCA 433 cells to a similar extent (Figure 5C). Neutralising IL-6R antibody (20 *μ*g ml^−1^) inhibited IL-6-induced migration to the level of control untreated cells and had a partial effect on the EGF-induced migration (45% wound closure compared to 60% in EGF-treated and 38% in control untreated cells) under similar conditions (Figure 5C). Consistent with that, EGF-induced IL-6 was partially inhibited with the same concentration of the antibody in OVCA 433 cells (Figure 5D) but completely inhibited in SKOV3 cells (data not shown). These results suggest that signalling from the IL-6R by secreted or cell-bound IL-6 produced in an autocrine manner in response to EGF affects the EGF-induced IL-6 secretion and migratory phenotype of ovarian cancer cells. These results suggest that the suppression of IL-6 secretion may have a significant inhibitory effect on the EGF-induced migration of OVCA 433 cells.

### Inhibition of the JAK2/STAT3 pathway does not affect EGF-induced morphology but blocks cell motility, EMT-induced N-cadherin and vimentin expression and IL-6 production

**Figure 6:**
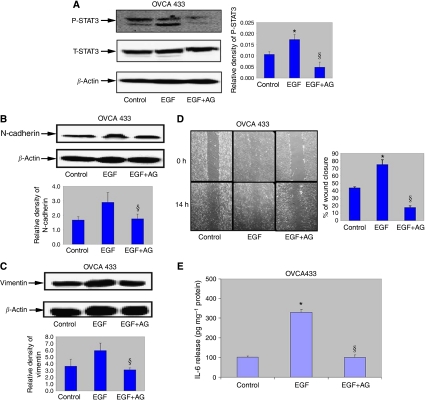
Effect of JAK2 inhibitor on STAT3 activation, EGF-induced N-cadherin, vimentin and cell migration. (**A**) Expression of P-STAT3 in OVCA 433 cells post-treatment with EGF and EGF+AG490 a specific JAK2 inhibitor. Cells were incubated in normal growth medium (control), medium treated with EGF (10 ng ml^−1^) or treated with EGF (10 ng ml^−1^) and AG490 (100 *μ*M) for 1 or 4 h. respectively. Western blot analysis was performed as described above for P-STAT3 followed by re-probing with STAT3 and *β*-actin. ^*^P<0.05, compared to control untreated cells and, ^§^compared to cells in the presence of EGF. Densitometry was performed as described above. (**B** and **C**) Expression of N-cadherin and vimentin 24 h post EGF in the absence and presence of AG490 in OVCA 433 cells. Western blot analysis for N-cadherin and vimentin was performed as described above. ^*^P<0.05, compared to control untreated cells, and ^§^compared to cells in the presence of EGF. (**D**) OVCA 433 cells were cultured on glass coverslips in either normal growth medium (control) or EGF-treated medium (10 ng ml^−1^) or in the presence of EGF (10 ng ml^−1^) and AG490 (100 *μ*M). Wounding assay was performed as described above. Images are representative of one independent experiment performed three times. Graphs represent mean % of wound closure from three independent experiments. Scale=300 *μ*m. ^*^P<0.05, compared to control untreated cells, and ^§^compared to cells in the presence of EGF. (**E**) IL-6 production in the presence and absence of EGF and EGF+AG490 was measured as described above. ^*^P<0.05, compared to control untreated cells, and ^§^compared to cells in the presence of EGF and AG490.

To confirm that the EGF-induced EMT is dependent on the downstream activation of JAK2/STAT3 from the IL-6R, we determined the effects of a specific JAK2 inhibitor (AG490) on the morphology and functional responses of cells treated with EGF for 24 h. Treatment of EGF-induced OVCA 433 cells with AG 490 (100 *μ*M) for 1 h resulted in the inhibition of STAT3 activation but there was no change in the total STAT3 expression (Figure 6A). Consistent with that, AG490 treatment in OVCA 433 cells resulted in the inhibition of N-cadherin and vimentin expression (Figure 6B and C) without any change in the expression of *β*-catenin (data not shown).

The effect of this pharmacological inhibition of JAK2 had no effect on the morphological phenotype of EGF-induced epithelial cells. Within 24 h in the presence of the inhibitor, the cells did not revert to an epithelial phenotype and maintained their fibroblast-like morphology (data not shown). In contrast, significant inhibition in cell motility was observed within 14 h (Figure 6D) in EGF-treated cells in the presence of AG490 (100 *μ*M), indicating that JAK2/STAT3 pathway may be involved in the EGF-induced motility of ovarian cancer cells. AG 490 treatment of OVCA 433 cells also resulted in the inhibition of EGF-induced IL-6 secretion (Figure 6E).

## Discussion

The acquisition of a motile function is correlated with a dramatic change in the cell physiology of most epithelia. Migrating cells no longer express epithelial characteristics and acquire mesenchymal properties. These include a loss of epithelial-like polygonal morphology, basal cell polarity and adhesive contacts, development of a fibroblast-like shape, reorganisation of cytoskeleton filaments, increased cell motility and induction of proteases for ECM degradation requisite for migration and invasion. Such profound changes have been reported previously as EMT, a cellular process manifested during embryo and organ morphogenesis (Hay and Zuk, 1995), wound healing (Tanaka *et al*, 2004), tumour progression (Theriault *et al*, 2007) and reproductive tract physiology (Demir *et al*, 2004). Some recent studies have implicated a role of STAT3 and EGFR in mediating EMT in cancer cells (Lo *et al*, 2007). Although the role of EMT in regulating metastasis in ovarian cancer cells is still not clear, overexpression of EGFR and constitutively active STAT3 have been shown in clinical specimens and ovarian cancer cell lines (Rosen *et al*, 2006). This warrants exploration into the significance of EGFR-mediated STAT3 activation in the dissemination of ovarian carcinoma. In this study, we show that EGF regulates many of the key processes that lead to mesenchymal transition in epithelial ovarian cancer cells and that autocrine signalling through the IL6-R/JAK2/STAT3 pathway is associated with these events and may regulate the metastasis of ovarian carcinoma.

We show that normal and benign ovaries lack overt JAK2 activation, which was only present in low- and high-grade ovarian carcinomas, despite JAK2 expression in normal ovaries, benign, borderline and histological grades of tumours. These findings suggest an important role for JAK2 activation in ovarian tumourigenesis. Low-to-moderate STAT3 activation was present in normal ovaries, benign and borderline tumours as well as low- to high-grade carcinomas but the status of activated STAT3 was significantly different between the normal, benign, borderline and malignant groups. These findings are consistent with the previous study, which has reported significantly higher STAT3 activation in carcinomas than in borderline tumours, even though no expression of activated STAT3 in normal ovarian surface epithelium was reported (Silver *et al*, 2004). The fact that JAK2 is only activated in ovarian carcinomas but not in normal ovaries or benign/borderline tumours suggests that the activation of STAT3 in normal ovaries and benign/borderline tumours may be independent of JAK2 activity and may depend on the intrinsic kinase activity of non-receptor cytoplasmic kinases such as SRC (Garcia *et al*, 2001) or the cytokine/growth factor stimulation of receptors with innate kinase activity (Badache and Hynes, 2001). None the less, the pathogenic role of JAK/STAT signalling pathway has been documented in cancer (Chan *et al*, 2004), the role of JAK2/STAT3 in the pathogenesis of ovarian cancer is still unknown. Therefore, the identification of JAK2 activation and increased STAT3 activation in ovarian carcinoma samples is a significant step towards answering this question.

To further evaluate the role of JAK2/STAT3 in the pathogenesis of ovarian cancer *in vitro,* studies were performed using SKOV3 and OVCA 433 as model ovarian cancer cell lines. We have previously shown that OVCA 433 and SKOV3 cell lines can be induced by EGF to undergo EMT-like phenotype characterised by the loss of epithelial-restricted E-cadherin and neutrophil gelatinase-associated lipocalin expression and a gain in the expression of mesenchyme-associated vimentin and increased motility (Lim *et al*, 2007). We now show that the activation of JAK2/STAT3 may be a pre-requisite for EGF-induced EMT in these ovarian cancer cell lines, as pharmacological inhibition of JAK2 in ovarian cancer cells not only led to an inhibition of STAT3 activation but also inhibition of EGF-induced EMT phenotypes. These findings highlight the potential significance of JAK2/STAT3 signalling in the acquisition of EMT-associated phenotypes of ovarian cancer cells, which may be vital for cancer progression and distant metastasis.

Genetic mutation or amplification of STAT proteins has not been identified in the pathogenesis of ovarian cancer. It has been proposed that constitutive STAT3 activation can result from aberrant EGFR signalling (Chan *et al*, 2004) and a significant correlation between high overall levels of P-STAT3 expression and overexpression of EGFR and HER-2/neu has been reported in ovarian cancer specimens (Rosen *et al*, 2006). Moreover, as only 30% of ovarian cancers express Her-2/neu or EGFR (Rosen *et al*, 2006), it can be suggested that the varying degrees of P-STAT3 expression exhibited by all the ovarian tumours analysed in our study may result due to the activation or upregulation of alternative cytokine or growth factor receptor pathways. Moreover, mutations in EGFR kinase domain have been shown to mediate STAT3 activation by IL-6 production in human lung carcinomas (Gao *et al*, 2007), suggesting that EGFR-like STAT3 activation can be mediated by IL-6R or GP130 receptors in an autocrine manner. To evaluate the secondary mediator-like response of IL-6 in response to ligand stimulation of EGFR, both OVCA 433 and SKOV3 cell lines were treated with EGF, and the mRNA expression of IL-6, IL-6R, LIF, LIFR and GP130 receptor were analysed within 1 h. The expression of IL-6 was significantly elevated in both ovarian cancer cell lines. The expression of LIF was enhanced to a lesser extent, and even though the relevant receptor expression was observed in both the cell lines, the expression of IL-6R*α* and GP130 was modestly increased in SKOV3 cells. To gain further insight into the mechanism of EGFR-induced cross talk with IL-6R pathway, we investigated the production of IL-6 in the serum-free medium of both the cell lines. Both OVCA 433 and SKOV3 enhanced the production of IL-6 in response to EGF stimulation and this enhancement was abrogated by neutralising IL-6R antibody and AG490 JAK2 inhibitor. Consistent with this fact, neutralising anti-IL6R partially inhibited EGF-induced migration in OVCA 433 cells, suggesting that EGF-induced migratory phenotype is dependent to a certain extent on the EGF-induced expression of IL-6 in certain ovarian cancer cells. A previous study has shown the expression of GP130 and LIFR*β* expression in ovarian carcinomas (Todd *et al*, 2002). From that perspective, IL-6R and GP130 family of receptors may form an extensive network of P-STAT3 signalling systems in ovarian carcinomas. However, it still remains to be determined if activation by EGF or somatic-activating mutations in the kinase domain of EGFR has any effect on the production of IL-6 or GP130 family of cytokines that can lead to the activated status of STAT3 in ovarian carcinomas.

In summary, this study provides strong evidence that activation of JAK2 and STAT3 directly or indirectly by autocrine induction may be one of the mechanism(s) that leads to ovarian cells becoming tumourigenic. A significant overall correlation between high levels of P-STAT3, EGFR and IL-6 expression has been reported in ovarian cancer specimens (Rosen *et al*, 2006). In this study, we further show that disruption of these pathways may result in the diminution of some of the EMT-associated phenotypes of ovarian cancer cells. Hence, diminution in EGFR/IL-6R/STAT3 signalling may represent novel therapeutic targets for pharmacological intervention in the management of ovarian cancer progression.

## Figures and Tables

**Table 1:** The expression of P-STAT3, T-STAT3, P-JAK2 and T-JAK2 in normal ovaries, benign, borderline and histological grades of ovarian tumours

**Antigen analysed**	**Normal ovaries**	**Benign tumours**	**Borderline tumours**	**Grade 1 tumours**	**Grade 2 tumours**	**Grade 3 tumours**	**Statistical analysis**
P-STAT3	0 (1)	0 (2)	1 (1)	0 (3)	0 (2)	1 (3)	*χ*^2^=32.74
	1 (4)	1 (5)	2 (3)	1 (4)	1 (5)	2 (4)	df=20, *P*=0.03
	2 (1)	2 (1)	3 (6)	2 (1)	2 (3)	3 (3)	
	3 (2)	4 (2)		4 (1)		4 (1)	
	4 (1)						
STAT3	0 (1)	1 (1)	1 (1)	4 (8)	3 (2)	2 (1)	*χ*^2^=28.69,
	2 (2)	2 (3)	2 (2)	5 (2)	4 (4)	3 (4)	df=25, *P*=0.276
	3 (3)	3 (2)	3 (3)		5 (4)	4 (4)	
	4 (3)	4 (2)	4 (3)			5 (2)	
		5 (1)	5 (2)				
P-JAK2	0 (4)	0 (6)	0 (2)	2 (1)	—	3 (4)	NA
				3 (1)			
T-JAK2	0 (3)	1 (1)	2 (2)	2 (1)	4 (2)	4 (1)	*χ*^2^=46.73,
	2 (1)	2 (1)	3 (3)	3 (2)	5 (8)	5 (8)	df=25, *P*=0.005
	3 (2)	3 (3)	4 (4)	4 (4)			
	5 (3)	4 (3)	5 (1)	5 (3)			
		5 (2)					

The extent of epithelial P-STAT3, T-STAT3, P-JAK2 and T-JAK2 expression was scored in a blind manner in normal ovaries and serous, endometrioid, mucinous, clear cell and mixed ovarian carcinomas of different pathological grades. Scoring was performed as 0 (⩽10%), 1 (⩾11–25%), 2 (⩾26–50%), 3 (⩾51–75%), 4 (⩾76–90%) and 5 (⩾91–100%) of immunoreactivity. Values in the parenthesis indicate number of patients evaluated for each antigen in each category. The epithelial distribution of antigens among normal and different tumour groups was statistically analysed using *χ*^2^ calculation.
