# Supplementary material for: Cross talk of signals between EGFR and IL-6R through JAK2/STAT3 mediate epithelial–mesenchymal transition in ovarian carcinomas
Source: Br J Cancer. 2008 Dec 16;100(1):134–44. doi: 10.1038/sj.bjc.6604794 (PMC2634691; doi:10.1038/sj.bjc.6604794)
Supplement: Supplementary Table [file 6604794x1.doc]

**Supplementary Table**

| ***Gene*** | ***Forward primer*** | ***Reverse primer*** |
| --- | --- | --- |
| *Cyclophilin* | CATCTGCACTGCCAAGACTGA | TTCATGCCTTCTTTCACTTTGC |
| *IL-6* | GGTACATCCTCGACGGCATCT | GTGCCTCTTTGCTGCTTTCAC |
| *LIF* | TGGTTCTGCACTGGAAACATG | AGTTGGTCTCCTGATCTGGTTCA |
| *IL-6Rα* | GCGAAAGGATGAAAGTGACCAT | AGACAAGCCCAGCAATGAAAA |
| *LIFRα* | GTGTTGCAGGTGCAGCAATT | AGAACCACAATCACTCCATACATC |
| *GP130* | TGCCCTTGGGAAGGTTACATC | GATACTAGACAGTTCCTCTGAGTTGATCA |
